# Validation of a portable pressure tile system for standing and stepping stability assessment in young and older adults

**DOI:** 10.1371/journal.pone.0351180

**Published:** 2026-07-01

**Authors:** Kasra Moradi, Brooke E. Peters, Luke Dillman, Chris A. McGibbon

**Affiliations:** 1 Faculty of Kinesiology, University of New Brunswick, Fredericton, New Brunswick, Canada; 2 Institute of Biomedical Engineering, University of New Brunswick, Fredericton, New Brunswick, Canada; Drexel University School of Biomedical Engineering Science and Health Systems, UNITED STATES OF AMERICA

## Abstract

Portable gait analysis technology for assessing mobility and balance among older adults in community environments remains an underutilized resource, often due to lack of field validation. To address this gap, we used a mixed-validation approach to evaluate a commercially available portable pressure tile system in terms of measurement accuracy: is it true enough to be believed; and sensitivity: can it detect age-related changes in balance and mobility? Thirty healthy adults were recruited in two cohorts of fifteen: YA = 19–64 years of age, and OA = 65 + years of age, to perform a battery of standing and locomotor tasks (static stand = SS, five-times sit-to-stand = STS, step-up/step-down task = SUSD with dominant and non-dominant lead) using a dual elevation, two-tile, StepScan™ pressure tile system placed upon in-floor mounted AMTI™ force platforms for simultaneous data acquisition. Common parameters for balance and stepping analysis were extracted from centre-of-pressure (CoP) kinematics from both systems and analyzed in terms of absolute agreement between systems (paired t-tests and Intra-Class Correlation, ICC(2,k)) and ability to discriminate by age group (independent t-tests and Pearson R^2^). Agreement between systems was high (<5mm error, ICC > .9) for most measures, and a small but statistically significant decline in balance and mobility performance was detected in the OA cohort compared to the YA cohort (R^2^ = .2, p < .05). We conclude that portable, modular, pressure tile systems such as StepScan™ are sufficiently accurate and sensitive for quantifying age-related changes in balance and mobility. Larger scale studies are needed to determine the potential for integrating this technology into routine clinical workflow.

## Introduction

Routine monitoring of gait and balance in later life could be useful for detecting changes in physical and cognitive functioning that lead to falls and/or indicate onset of neurodegenerative disease [1]. Centre of pressure (CoP) excursion during standing and locomotor tasks is commonly used for deriving key indicators of balance and stepping stability [2,3], particularly in aging populations where the risk of falls increases. A distinct advantage of CoP-based measurement is simpler data acquisition and analysis compared to body-based (centre of mass, CoM) measurement tracking with motion analysis cameras or wearable sensors, both of which require a model having many assumptions regarding the CoM location and/or its kinematic behaviour [4–6]. CoP excursion is easily obtainable from in-floor force platforms that represent the gold standard in research laboratory [7], but are mostly inaccessible for routine clinical assessment of community dwelling seniors.

A critical need in this space are portable turnkey technologies that can be deployed in clinics and community settings for objective and comprehensive mobility and balance assessment. Portable pressure sensing floor tiles (StepScan Technologies, Inc., Charlottetown, PEI, Canada) offer a potential solution for routine mobility and balance assessment in the home or community, as they consist of modular 60 cm x 60 cm tiles (5 mm sensor resolution), that can be used individually or connected in any combination to measure foot pressure distribution as well as foot-floor interactions (CoP trajectory) during movement tasks.

Previous studies have used the StepScan™ as a comparator for validating markerless motion capture systems in walking gait [8,9]. In a preliminary study with fifteen young adults [10] we reported CoP x,y coordinate accuracy data for the StepScan™ system relative to AMTI force plates and showed that once bias was removed from the CoP trajectories, the error (i.e., difference in raw CoP x,y coordinates between StepScan™ tile on top of AMTI plate) decreased from >20mm to <5mm for standing and stepping activity. This study showed that StepScan™ pressures tiles may be suitable for applications where relative excursion is germane to the observer, such as parameterization (i.e., reduction) of the CoP x,y trajectories into common measures of balance and stability, as detailed above.

However, to our knowledge, StepScan™ tiles have not been validated for CoP-based measures of stability during standing and locomotor tasks, nor has the ability of the technology to detect age-related changes in standing and locomotor stability been established. Hence the purpose of the present work was to explore if CoP-derived balance and stability parameters from the pressure tiles and gold-standard in-floor force platforms agree across a pooled sample of fifteen young (from [10]) and fifteen older adults (N = 30), and whether the StepScan™ derived stability parameters are sensitive enough to detect balance decline with aging. To the latter point, there are numerous potential outcomes measures for representing standing and locomotor stability; a related goal was to develop and test a protocol that included: quiet standing, repeated chair rise, and a repeated stepping task, that can be completed with two pressure tiles in a limited space environment (such as a clinic or home). The specific aims of the study were:

1) to validate StepScan™ CoP measurement derived balance and stability parameters using in-floor force plates (AMTI, Amherst, MA) which represent the industry gold-standard in human motion capture; and
2) to evaluate whether balance and stepping stability parameters derived from StepScan™ CoP can discriminate between healthy young and older adults.

## Methods

The study took place between 2023–2025 in a motion analysis laboratory at the Centre for Adaptive Rehabilitation Engineering, located at the Institute of Biomedical Engineering at the University of New Brunswick, Canada. The protocol was reviewed and approved by the university Research Ethics Board and all volunteers provided written informed consent prior to participating.

### Participants

A convenience sample of 30 healthy adults recruited from the community participated in the study. Sample size was determined using G*Power (version 3.1.9.7) with alpha = .05, power = .8, and effect size equal to the CoP threshold error of 5 mm divided by the expected standard deviation of 10 mm (d = .5). The required sample size was N = 27. To protect against data loss or study withdrawals, we planned for recruiting N = 30, which was divided into two cohorts of n = 15 (young and older adults) as detailed below.

Participants were recruited through word of mouth or by responding to posters placed at local community groups. We first recruited and tested 15 younger adults (YA = 19–64 yrs) to ensure a) the testing protocol was safe and feasible and b) that the pressure tiles possessed adequate accuracy for deriving stability measures (see [10]). With no adverse events and encouraging results from [10] we next recruited and tested 15 older adults (OA = 65 yrs or older) to achieve a pooled sample of N = 30. The recruitment period was March 8, 2024, to February 8, 2025. Inclusion criteria were being an adult aged 19 or older and exclusion criteria were any acute or chronic conditions that would affect mobility (stepping, walking) and balance or having conditions with contraindications to physical exercise. The Get Active Questionnaire is commonly used for such screening [11] and was used in our study to screen potential volunteers for the above mentioned exclusion criteria.

### Procedures

#### Motion laboratory set-up.

**Fig 1:**
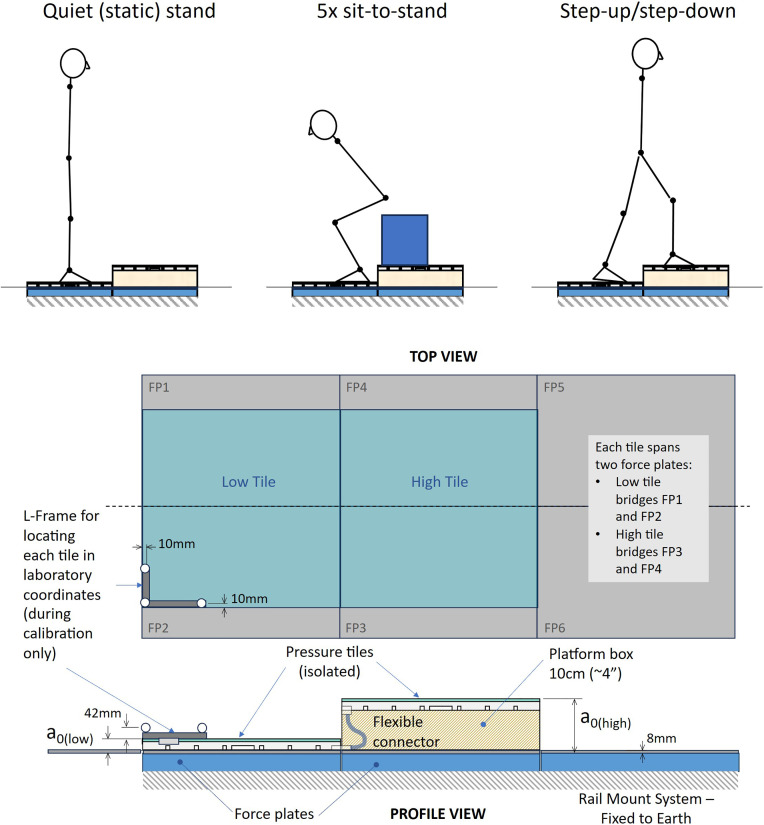
Top panel: Standing, Chair rise and Stepping task on the experimental platform. Bottom panel: Experimental set-up of pressure tiles on top of force platforms. Adapted from [10].

The motion analysis laboratory was equipped with 24 wall-mounted optoelectronic motion tracking cameras (Optitrak, NaturalPoint Inc., Corvallis, OR) and six in-floor force platforms (AMTI 4060, AMTI, Amherst MA) arranged in a standard 2x3 configuration, as shown in Fig 1 (gray rectangles). Prior to each data collection session, two StepScan™ tiles (teal rectangles) were placed as shown on top of the force plates. The “low” tile was placed directly on force plates #1 and #2, and the “high” tile was placed on top of a rigid wooden box with flat bottom having a height of 10 cm (4 inches) which sat stationary on force plates #3 and #4. Tile positions were monitored throughout the testing, and while not detailed here, showed that the tiles could be considered rigidly attached to the plates and thus standard equations for locating the CoP on the contact surface of the tiles could be employed.

Because the pressure tiles were 60 cm long x 60 cm wide, and two adjacent force plates measured 60 cm long x 80 cm wide, the low and high tiles could be carefully aligned in the A/P (X) direction with the edges of the forces plates as a reference. This ensured the low tile always bridged force plates #1 and #2, and the high tile (and box) always bridged force plates #3 and #4. A calibration frame (L-Frame shown in Fig 1, lower panel) was positioned on the right-hand lower corner of each tile to precisely register its local coordinate (x-y-z) system in the global laboratory coordinate (X-Y-Z) system.

#### Data collection protocol.

Upon arrival at the laboratory, participants changed from their street clothes into clothing appropriate for data collection, and motion capture markers were placed on the participants shoes and pelvis. Participants first warmed up by walking around the laboratory on an indoor walking track, where heel marker data were used to estimate the participant’s preferred walking pace. Participants were then asked to perform a series of activity trials consistent with three common activities performed for clinical assessment of balance and mobility in older adults: quiet standing, repeated sit-to-stand, and a paced stepping task (Fig 1, top panel). All trials were collected on the pressure tiles (100 Hz), which were time synchronized with the force plates (1000 Hz, down sampled to 100 Hz).

*Quiet (static) standing (SS)*: Participants performed three sets of quiet standing tests with their feet shoulder-distance apart and their eyes open. Each standing test was conducted for 10 seconds. One test was performed while standing on FPs 5 and 6 (no tiles), one while standing on the Low tile (on top of FP 1 + 2), and one while standing on the High tile (on top of FP 3 + 4).

*Five times Sit-to-Stand (STS)*: Participants performed five repeated sit-to-stands from an armless and backless chair, while their feet were placed on the Low tile (on top FP 1 + 2) and seat (heavy wooden box) positioned on the High tile (on top of FP 3 + 4).

**Fig 2:**
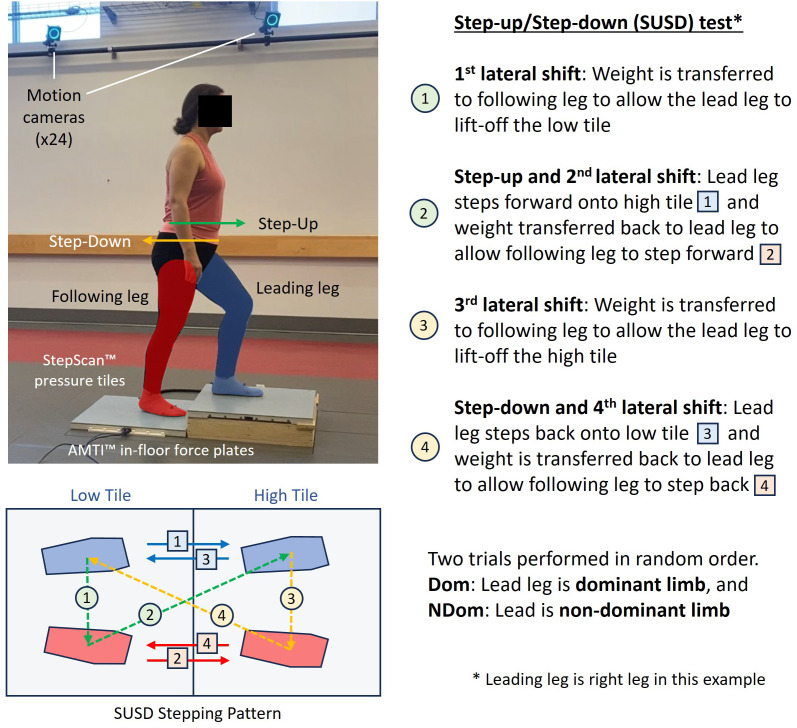
Step-up/Step-down (SUSD) test, with leading leg shaded in blue and lagging leg shaded in red. Two trials are completed, once with the dominant leg leading and once with the non-dominant leg leading. Adapted from [10].

*Step-up/Step-down (SUSD)*: Participants were first asked to identify their dominant limb by declaring which leg they would use to kick an imaginary ball rolled toward them. This was necessary because the SUSD task required only one leg to perform all the upward motion during the task (Fig 2). To control for potential order effects, the starting leg (dominant or non-dominant) was randomized across participants.

1. SUSD Dominant: With the metronome set at the participant’s preferred pace, the participant started the SUSD protocol by stepping up with their dominant limb and continued stepping up and back down for 30 seconds.
2. SUSD Non-dominant: With the metronome set at the participant’s preferred pace, the participant started the SUSD protocol by stepping up with their non-dominant limb and continued stepping up and back down for 30 seconds.

Participants were allowed to rest for 1–2 minutes (as needed) between trials.

#### Data processing and analysis.

Pressure tile and force plate data were processed using custom Matlab script. Briefly, pressure data were analyzed to extract centre of pressure (CoP) coordinates registered in each tile’s coordinate system and transformed (from the L-frame calibration) into global coordinates. Because each of the pressure tiles sat upon two force plates, effectively bridging them, the force plate signals were combined by summing the forces and moments of the two adjacent plates. As such, FPs 1 + 2 corresponded to the Low tile, and FPs 3 + 4 corresponded to the High tile.

Force plate CoP coordinates were calculated in global coordinates using the “a_0_” term (see Fig 1) as the distance between the force plate surface and the plantar surface, defined by the height of each tile as determined during calibration. Force plate data were down sampled to 100 Hz to match the StepScan™ sample rate of 100 Hz. Signals were time synchronized by registering the “on” frame where the vertical force from the force plate and tile first exceeded 10N, when the participant stepped onto the tile at the beginning of each data collection trial.

#### Balance/stability measurement analysis.

Outcome variables, relevant units and performance targets are summarized in Table 1. The three activities selected for study were as follows:

**Table 1:** Outcome variables and descriptors.

Variable	Description	Test value*
*Static (quiet) standing*		
SDx, SDy	Standard deviation of CoP in x and y directions.	5mm
Dmaj, Dmin	Major and minor diameters of an ellipse fit to CoP trajectory.	5mm
Vel	Mean resultant velocity of CoP.	5mm/s
*5x Sit-to-Stand*		
APvar	Standard deviation of CoP excursion in x direction.	5mm
MLvar	Standard deviation of CoP excursion in y direction.	5mm
Var	Combined APvar and MLvar.	5mm
tVar	Standard deviation in chair sit-to-stand time.	.01s
*Step-up/Step-down*		
Dom-Up	Standard deviation of CoP excursion in y direction (step width) for Step-up, Dominant leg is leading.	5mm
NDom-Up	Standard deviation of CoP excursion in y direction (step width) for Step-up, Non-dominant leg is leading.	5mm
Dom-Dn	Standard deviation of CoP excursion in y direction (step width) for Step-down, Dominant leg is leading.	5mm
NDom-Dn	Standard deviation of CoP excursion in y direction (step width) for Step-down, Non-dominant leg is leading.	5mm

* Test value for single-sample t-test of error magnitude.Statistical Analyses.

*Quiet (static) standing*: Key parameters that can be derived from the CoP X-Y trajectory, and that are of interest for assessing standing balance [2], include the standard deviation in anterior/posterior (A/P) and medial/lateral (M/L) directions [2,12], fitting an ellipse to enclose 95% of the trajectory and quantifying the major and minor diameters, and calculating the mean velocity of the CoP [2,3].

*Five times Sit-to-Stand (STS)*: The chair-rise maneuver imposes high torque demands on the knees and hips, making it sensitive to age-related declines in lower-limb strength and balance control that may not be detected in static standing tasks [13]. In particular, spatiotemporal parameters derived from CoP and CoM trajectories—such as prolonged rise time, delayed weight-shift initiation, and reduced vertical CoM velocity—have been associated with impaired postural transitions and increased fall risk in older adults [14]. Therefore, extracted parameters include variability in A/P and M/L CoP excursion and variability of STS time interval during the repeated STS.

*Step-up/Step-down (SUSD)*: Overground stationary stepping tasks, such as the SUSD have been studied [15,16] where stepping stability was quantified as variability in the repeated pattern of CoM trajectory during the test [17]. The CoM produces a relatively smooth trajectory, whereas the CoP has discontinuities and/or sharp transitions, but which can be parameterized in terms of variability in A/P excursion of the CoP (i.e., step length variability) and variability in M/L excursion of the CoP (i.e., step width variability). Step length during the SUSD, however, is fixed to some extent by the platform set-up and dimensions, while step width during the SUSD is freer to vary; thus we focus on M/L CoP excursion using step width variability as the stability metric for SUSD up-stepping, and down-stepping, for dominant and non-dominant tests.

Research question #1: Do stability parameters derived from pressure tiles agree with those derived from the industry “gold standard” (in-floor) force plates?

Agreement between calculated stability parameters from pressure tiles and from the force plates was evaluated with t-tests, Intraclass Correlation Coefficient (ICC(2,k) model) analysis [18], and Kendall’s tau-b analysis for hetroscedasticity [19]. Because the StepScan™ tiles have a measurement resolution of 5 mm (each pressure cell is a 5 mm square) the target accuracy for the t-test comparisons was set to 5 mm. To ensure robust agreement is achieved, we set the ICC criteria as the lower-bound on the 95% confidence interval being greater than 0.7. Finally, Kendall’s *tau-β* correlation which quantifies the independence of the measurement error on measurement values, should be non-significant.

As such, there were three hypotheses tested:

**Hypothesis H1a**: *The mean difference between stability parameters derived from pressure tiles and force plates for SS, STS and SUSD tests will be 5 mm or less*. ***Hypothesis rejection criteria****: The mean difference is significantly more than 5 mm*.

**Hypothesis H1b**: *ICC analysis between stability parameters derived from pressure tiles and force plates will yield an ICC with lower bound of the 95% confidence interval ≥ .7*. ***Hypothesis rejection criteria****: The lower-bound of the 95% confidence interval is < .7.*

**Hypothesis H1c**: *Limits of agreement analysis (via Bland-Altman plots* (20)*) between the pressure tiles and force plates derived stability parameters, will have a non-significant (p > .05) Kendall’s tau-*β *correlation between difference scores and mean of scores.* ***Hypothesis rejection criteria****: Tau-*β *correlation is positive (τ*_*β*_ *> .2) and significant (p < .05)*.

Research question #2: Are stability parameters derived from pressure tiles sufficiently sensitive to detect age-related decline in balance?

It is well understood and documented in the literature that there are age-related changes in stability during standing and gait in the later years of life, even for older adults that are otherwise healthy and fit [20]. Generally, these changes are not due to pathology or impairment but rather reflect the age-related decline in sensory organization (i.e., vestibular-visual-somatosensory) in vertebrate animals [21]. As such these age-related changes can be subtle and often require a variety of investigative approaches to gain a clear picture of whether the changes are meaningful. The purpose of Aim 2 was to determine if standing and locomotor stability measures from the pressure tiles are sensitive enough to detect age-related declines in balance among otherwise healthy adults.

**Hypothesis H2a**: *There will be a significant between-group difference (p < .05) in stability measures where older adults will be less stable during standing and locomotor activity than younger adults.* ***Hypothesis rejection criteria****: The null hypothesis is accepted that no significant difference is found (p > .05)*.

**Hypothesis H2b**: *There will be a significant (p < .05) and meaningful (r > .3) correlation between the age of participant and their stability scores.* ***Hypothesis rejection criteria****: The correlation is non-significant (p > .05) or explains too little variance (r < .3).*

## Results

### Participants

Thirty healthy volunteers between the ages of 19 and 86 participated in the study, as summarized in Table 2. There were no significant differences in height, weight, body mass index, or walking speed between the two groups.

**Table 2:** Participant characteristics and starting limb randomization for SUSD.

Group† (N)		Age (yrs)		Mass (kg)		Height (m)		BMI (kg/m^2^)		Speed (m/s)		Start limb*	
		Mean	SD	Mean	SD	Mean	SD	Mean	SD	Mean	SD	D	ND
YA	F (9)	27.7	8.8	62.2	12.1	1.66	.09	22.4	2.79	1.15	.20	4	5
	M (6)	26.8	7.4	74.8	6.9	1.77	.07	24.2	3.48	1.16	.19	3	3
OA	F (11)	73.2	5.5	61.8	11.1	1.59	.06	24.3	3.81	1.06	.15	5	6
	M (4)	71.5	2.9	94.1	21.8	1.82	.08	28.3	5.17	1.06	.21	3	1

* D = Dominant limb; ND = Non-dominant limb; † F = Female; M = Male.

### Hypothesis tests

Research Question #1: Do stability parameters derived from pressure tiles agree with those derived from the industry “gold standard”, in-floor force plates?

Table 3 shows results of H1a, H1b, and H1c tests for the various stability parameters calculated for each of the three activities.

**Table 3:** Pressure tile (PT) versus force plate (FP) for balance and stability parameters during the static stand trials, 5-times sit-to-stand (5xSTS) trials, and step-up/step-down (SUSD) trials.

Stability Param.	Device	Mean	SD	H1a: One-sample t-test Diff < 5 mm		H1b: Intraclass Correlation stats		H1c: Kendall’s tau-b stats	
				*Diff (SD)*	*p-value*	*ICC*_*(2,k)*_	*[95% CI]*	*Tau-b*	*p-value*
***Standing Stability Parameters***									
SDx (mm)	PT	8.52	4.41	0.59 (1.13)	<.001†	.977	[.942 -.990]	.154	.232
	FP	7.97	3.88						
SDy (mm)	PT	6.01	3.06	0.44 (1.15)	<.001†	.959	[.910 -.981]	.149	.246
	FP	5.57	3.03						
Dmaj (mm)	PT	17.8	10.12	0.89 (4.64)	.003†	.936	[.866 -.969]	.136	.293
	FP	16.9	8.71						
Dmin (mm)	PT	8.34	4.23	0.17 (1.45)	<.001†	.968	[.933 -.985]	.021	.872
	FP	8.17	3.92						
Vel (mm/s)	PT	16.1	6.89	0.17 (2.07)	<.001†	.978	[.954 -.989]	.067	.605
	FP	15.9	7.01						
***5xSTS CoP Excursion Variability***									
APvar (mm)	PT	19.4	6.76	3.87 (3.71)	.119	.816	[.120 -.939]	.275	.040*
	FP	15.5	5.55						
MLvar (mm)	PT	14.4	5.50	1.22 (3.81)	<.001†	.826	[.626 -.919]	.132	.323
	FP	13.1	4.58						
Var (mm)	PT	24.6	7.36	3.85 (4.10)	.150	.825	[.244 -.940]	.291	.030*
	FP	20.7	6.00						
tVar (s)	PT	.233	.202	−.011 (.036)	.946	.992	[.983 -.996]	.069	.607
	FP	.243	.216						
***SUSD Step Width Variability***									
Dom-Up (mm)	PT	19.7	5.61	−0.21 (1.48)	<.001†	.982	[.962 -.992]	−.016	.906
	FP	19.9	5.60						
Dom-Dn (mm)	PT	27.0	7.77	−2.01 (2.21)	<.001†	.968	[.796 -.990]	−.310	.018*
	FP	29.0	8.68						
NDom-Up (mm)	PT	17.2	5.11	0.30 (1.36)	<.001†	.981	[.960 -.991]	.113	.388
	FP	16.9	4.97						
NDom-Dn (mm)	PT	22.8	6.10	0.10 (1.64)	<.001†	.981	[.960 -.991]	.034	.793
	FP	22.7	5.82						

* Significant Kendall’s tau-b at alpha =.05.

† Significant one-tailed hypothesis test at alpha = .05.

H1a tested whether the difference between parameter estimates from pressure tiles (PT) and force plates (FP) exceeded the specified threshold of acceptance (5 mm, 5 mm/s, etc.). Results showed mean differences significantly below the threshold, or not different from the threshold; no test showed differences significantly exceeding 5 mm.

H1b tested whether the ICC values had a lower bound on the 95% confidence interval that exceeded 0.7. Results showed that only the 5xSTS trials had any questionable agreement levels. A/P CoP excursion variability (APvar) and the combined A/P and M/L CoP excursion variability (Var) had ICC lower bounds that fell well below 0.7 (.12 and.24, respectively), and M/L CoP excursion variability fell just below the 0.7 threshold (.63); all other variables had high levels of agreement (>.9).

**Fig 3:**
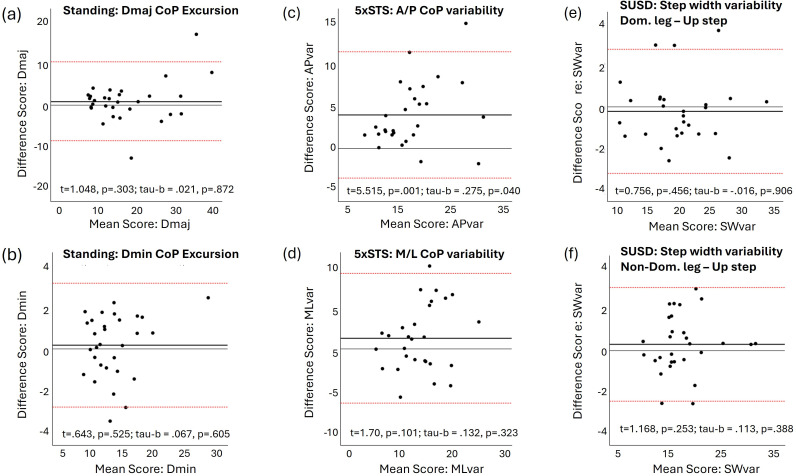
Bland-Altman plots for key balance and stability metrics: a) and b) show Dmaj and Dmin statistics for static standing, respectively; c) and d) show A/P and M/L CoP excursion variability statistics for the 5xSTS activity, respectively, and; e) and f) show step width variability statistics for the dominant and non-dominant step up leg of the SUSD, respectively.

H1c tested whether the difference between means was related to the magnitude of the means (heteroscedastic). Results showed that 5xSTS parameters APvar and Var did not pass the Kendall’s tau-b criteria, as well as the step width variability during the down phase of the Dominant lead SUSD (Dom-Dn). All other variables were found to be homoscedastic. Fig 3 shows representative Bland-Altman plots for key parameters.

Research question #2: Are stability parameters derived from pressure tiles sufficiently sensitive to detect age-related decline in balance?

Table 4 shows results of H2a comparing the StepScan™ outcomes between young (Y, n = 15) and older (O, n = 15) adults, and H2b evaluating the correlation between age in years and magnitude of the various stability measures.

**Table 4:** Relationship between age and StepScan derived balance and stability parameters for the static stand trials, 5-times sit-to-stand (5xSTS) trials, and step-up/step-down (SUSD) trials.

Stability Param.	Age Group	Mean	SD	H2a: Independent samples t-test			H2b: Pearson correlation w/age	
				*t*	*df*	*p*	*r*	*p*
***Standing Stability Parameters***								
SDx (mm)	Y	7.13	3.43	−1.796	28	.042†	.347	.030†
	O	9.91	4.93					
SDy (mm)	Y	4.93	1.84	−2.038	20.6*	.027†	.321	.042†
	O	7.09	3.68					
Dmaj (mm)	Y	13.5	5.91	−2.557	20.7*	.009†	.437	.008†
	O	22.1	11.7					
Dmin (mm)	Y	7.87	2.61	−.601	28	.276	.112	.278
	O	8.81	5.46					
Vel (mm/s)	Y	13.5	5.48	−2.139	28	.021†	.357	.026†
	O	18.6	7.39					
***5xSTS CoP Excursion Variability***								
APvar (mm)	Y	19.5	6.97	.102	26	.460	.017	.465
	O	19.3	6.79					
MLvar (mm)	Y	13.8	5.43	−.519	26	.304	.067	.367
	O	14.9	5.72					
Var (mm)	Y	24.3	7.54	−.164	26	.436	.041	.418
	O	24.8	7.47					
tVar (s)	Y	.156	.084	−2.112	15.8*	.026†	.337	.040†
	O	.309	.257					
***SUSD Step Width Variability***								
Dom-Up (mm)	Y	19.9	6.08	−.157	26	.438	.094	.317
	O	19.9	5.27					
Dom-Dn (mm)	Y	26.8	8.00	−.132	27	.448	.060	.378
	O	27.2	7.81					
NDom-Up (mm)	Y	15.1	3.22	−2.616	27	.007†	.450	.007†
	O	19.6	5.81					
NDom-Dn (mm)	Y	21.5	4.65	−1.199	27	.121	.191	.160
	O	24.2	7.27					

* Degrees of freedom (df) adjusted for unequal variances from Levene’s test.

† Significant one-tailed hypothesis test at alpha = .05.

Significant differences were observed between younger and older adults in several static standing stability measures—SDx (p = .042), SDy (p = .027), Dmaj (p = .009) and Vel (p = .021)—while Dmin did not differ significantly (p = .276). Age was also significantly and positively correlated with SDx (r = .347, p = .030), SDy (r = .321, p = .042), Dmaj (r = .437, p = .008) and Vel (r = .357, p = .026), but not with Dmin (r = .112, p = .278).

For 5xSTS trials, only tVar (peak-to-peak sit-to-stand time variability) was significantly greater in older adults (*p* = .026), indicating increased inconsistency in the timing of sit-to-stand transitions with age. However, APvar**,** MLvar**,** and combined variability (Var) did not differ significantly between younger and older adults (*p* > .05), suggesting that spatial variability in CoP excursion during sit-to-stand tasks is not markedly affected by age.

For the SUSD step width variability measures, only the non-dominant foot during the step-up condition (NDom-Up) showed a significant difference between age groups, with older adults demonstrating greater variability (*p* = .007). No significant differences were observed for the dominant foot step-up (Dom-Up; *p* = .438), dominant foot step-down (Dom-Dn; *p* = .448), or non-dominant foot step-down (NDom-Dn; *p* = .121) conditions.

## Discussion

Mobility and balance impairment among older adults can lead to significant adverse outcomes such as fall related injuries, hospitalizations, and deaths [22]. Guidelines for monitoring mobility and balance among community-dwelling seniors have largely focused on observational or simple timed measures of function in the context of falls-risk screening [23,24]. Although falls-risk screening algorithms can be sensitive to self-report in the higher-risk category (self-reported prior fall in the last year being the best predictor) [25] these tools were not designed as comprehensive mobility and balance assessments protocols for those in the moderate-risk category or without a fall history. In this cohort the consensus among experts is that there is no one test or metric that can predict falls in older adults [26,27], particularly given the multi-factorial influences of frailty and cognitive decline [28–30]. Therefore, how to predict (and prevent) a first fall or other health event (disease onset) is arguably an even more paramount and challenging question to answer.

Given the growing need for reliable, accessible gait and balance assessment tools [1], our study fills a gap by validating CoP measurements from a portable pressure tile system against the gold standard in-floor mounted force plate system, as well as analyzing its ability to discriminate between young and older healthy adults as a demonstration of its potential to be sensitive to subtle changes in stability.

### Measurement performance

Statistical analysis revealed high agreement between StepScan™ and AMTI for key CoP-derived metrics, including standard deviation in the medial-lateral (SDx) and anterior-posterior (SDy) directions, major and minor axis lengths of the ellipse (Dmaj, Dmin), and mean CoP velocity. Average differences between systems was often on the order of 1–3 mm, which is within the StepScan™ measurement resolution of 5 mm. What this means in practice is that the accuracy of relative movement of the CoP is within the 1-pixel precision of the sensor matrix (5 mm). These results confirm the suitability of StepScan™ for measuring postural control under static conditions and also provides a threshold of detection for designing experiments where a minimally important clinical difference or change (often much greater than 5 mm) is the effect being measured.

For dynamic tasks like the 5xSTS and SUSD, parameters derived from M/L CoP excursion had the best agreement between StepScan™ and AMTI (compared to A/P metrics), which included all four “step width” stability metrics extracted from the Dominant and Non-dominant SUSD tests. ICC values (mostly >0.9) and non-significant Kendall’s tau-b (mostly p > .05), support the robustness of StepScan™ for measurement of static and dynamic stability parameters.

### Measurement sensitivity

Although there were no differences in height, weight or walking speed, between the young and older adult groups, older adults in our study demonstrated significantly greater variability in CoP excursions, consistent with previous findings that indicate increased postural sway and reduced balance control with age. For example, Park et al. (2016) and Stins & Roerdink (2018) reported that older adults exhibit significantly greater mediolateral CoP excursions and increased sway velocity, both of which are associated with increased fall risk (23,24), and may be independent of walking speed. This is important given the singular focus on “gait speed” in falls risk assessment algorithms [23,24].

In our study, Dmaj (R² = 0.191) and CoP velocity (R² = 0.128) for standing balance tests, tVar (R² = 0.114) for sit-to-stand tests, and NDom-Up (R² = 0.20) showed positive correlations with age. Although statistically significant, the explanatory power (11–20%) suggests that while aging contributes to postural instability, it is not the only factor. These findings align with the multifactorial model of postural decline, which implicates deterioration in visual, vestibular, and somatosensory inputs, as well as slower neuromuscular responses [25]. The ability to detect these subtle variations with portable pressure tiles underscores the potential utility of this technology as a practical screening tool for early balance deterioration in older adults.

### Limitations

Older adult participants in our study were in good physical health and therefore may not reflect the general population of seniors. Although no statistically significant difference could be detected between young and old participants in terms of height and body weight, larger samples may prove to detect such differences, and as such should also be considered in future studies.

## References

[1] Cullen S, Montero-Odasso M, Bherer L, Almeida Q, Fraser S, Muir-Hunter S, et al. Guidelines for Gait Assessments in the Canadian Consortium on Neurodegeneration in Aging (CCNA). Can Geriatr J. 2018;21(2):157–65. doi: 10.5770/cgj.21.298 29977431 PMC6028168

[2] Quijoux F, Nicolaï A, Chairi I, Bargiotas I, Ricard D, Yelnik A, et al. A review of center of pressure (COP) variables to quantify standing balance in elderly people: Algorithms and open-access code. Physiol Rep. 2021;9(22):e15067. doi: 10.14814/phy2.15067 34826208 PMC8623280

[3] Doyle RJ, Hsiao-Wecksler ET, Ragan BG, Rosengren KS. Generalizability of center of pressure measures of quiet standing. Gait Posture. 2007;25(2):166–71. doi: 10.1016/j.gaitpost.2006.03.004 16624560

[4] Tisserand R, Robert T, Dumas R, Chèze L. A simplified marker set to define the center of mass for stability analysis in dynamic situations. Gait Posture. 2016;48:64–7. doi: 10.1016/j.gaitpost.2016.04.032 27477710

[5] Nicolai A, Audiffren J. Estimating center of mass trajectory in quiet standing: A review. Annu Int Conf IEEE Eng Med Biol Soc. 2019;2019:6854–9. doi: 10.1109/EMBC.2019.8857888 31947415

[6] Banks A, He R, Dillman L, McGibbon C, Sensinger J. A Comparison of force-plate based center of mass estimation algorithms. IEEE Int Conf Rehabil Robot. 2022;2022:1–5. doi: 10.1109/ICORR55369.2022.9896525 36176157

[7] Błaszczyk JW. The use of force-plate posturography in the assessment of postural instability. Gait Posture. 2016;44:1–6. doi: 10.1016/j.gaitpost.2015.10.014 27004624

[8] McGuirk TE, Perry ES, Sihanath WB, Riazati S, Patten C. Feasibility of markerless motion capture for three-dimensional gait assessment in community settings. Front Hum Neurosci. 2022;16:867485. doi: 10.3389/fnhum.2022.867485 35754772 PMC9224754

[9] Riazati S, McGuirk TE, Perry ES, Sihanath WB, Patten C. Absolute Reliability of gait parameters acquired with markerless motion capture in living domains. Front Hum Neurosci. 2022;16:867474. doi: 10.3389/fnhum.2022.867474 35782037 PMC9245068

[10] Moradi K, McGibbon CA. Validation of Centre of Pressure Trajectory from a Portable Gait System. CMBEC47/ACCESS26 Proc., Fredericton, NB, 2025. https://proceedings.cmbes.ca/index.php/proceedings/article/view/1247/1094

[11] Canadian Society for Exercise Physiology. Get Active Questionnaire. https://csep.ca/2021/01/20/pre-screening-for-physical-activity/. 2021.

[12] Paillard T, Noé F. Techniques and methods for testing the postural function in healthy and pathological subjects. Biomed Res Int. 2015;2015:891390. doi: 10.1155/2015/891390 26640800 PMC4659957

[13] Alexander NB, Gross MM, Medell JL, Hofmeyer MR. Effects of functional ability and training on chair-rise biomechanics in older adults. J Gerontol A Biol Sci Med Sci. 2001;56(9):M538-47. doi: 10.1093/gerona/56.9.m538 11524445

[14] Scarborough DM, McGibbon CA, Krebs DE. Chair rise strategies in older adults with functional limitations. J Rehabil Res Dev. 2007;44(1):33–42. doi: 10.1682/jrrd.2005.08.0134 17551856

[15] McPartland MD, Krebs DE, Wall C 3rd, New Collective Author. Quantifying ataxia: Ideal trajectory analysis--a technical note. J Rehabil Res Dev. 2000;37(4):445–54. 11028700

[16] Hudson CC, Krebs DE. Frontal plane dynamic stability and coordination in subjects with cerebellar degeneration. Exp Brain Res. 2000;132(1):103–13. doi: 10.1007/s002219900291 10836640

[17] McGibbon CA, Krebs DE, Wagenaar R. Stepping stability: Effects of sensory perturbation. J Neuroeng Rehabil. 2005;2:9. doi: 10.1186/1743-0003-2-9 15921515 PMC1180849

[18] Shrout PE, Fleiss JL. Intraclass correlations: Uses in assessing rater reliability. Psychol Bull. 1979;86(2):420–8. doi: 10.1037//0033-2909.86.2.420 18839484

[19] Myles PS, Cui J. Using the Bland-Altman method to measure agreement with repeated measures. Br J Anaesth. 2007;99(3):309–11. doi: 10.1093/bja/aem214 17702826

[20] Colón-Emeric CS, Whitson HE, Pavon J, Hoenig H. Functional decline in older adults. Am Fam Physician. 2013;88(6):388–94. 24134046 PMC3955056

[21] Goble DJ, Coxon JP, Wenderoth N, Van Impe A, Swinnen SP. Proprioceptive sensibility in the elderly: Degeneration, functional consequences and plastic-adaptive processes. Neurosci Biobehav Rev. 2009;33(3):271–8. doi: 10.1016/j.neubiorev.2008.08.012 18793668

[22] Bhasin S, Gill TM, Reuben DB, Latham N, Peduzzi P. Preventing Serious Falls among Older Adults -- The STRIDE Study. Washington (DC). 2022. doi: 10.25302/06.2022.MOUNIH.2013001 39869722

[23] Mark JA, Loomis J. The STEADI toolkit: Incorporating a fall prevention guideline into the primary care setting. Nurse Pract. 2017;42(12):50–5. doi: 10.1097/01.NPR.0000525720.06856.34 29176440

[24] Montero-Odasso M, van der Velde N, Martin FC, Petrovic M, Tan MP, Ryg J, et al. World guidelines for falls prevention and management for older adults: A global initiative. Age Ageing. 2022;51(9):afac205. doi: 10.1093/ageing/afac205 36178003 PMC9523684

[25] Ritchey K, Olney A, Chen S, Phelan EA. STEADI self-report measures independently predict fall risk. Gerontol Geriatr Med. 2022;8:23337214221079222. doi: 10.1177/23337214221079222 35647219 PMC9133870

[26] Lusardi MM, Fritz S, Middleton A, Allison L, Wingood M, Phillips E, et al. Determining risk of falls in community dwelling older adults: A systematic review and meta-analysis using posttest probability. J Geriatr Phys Ther. 2017;40(1):1–36. doi: 10.1519/JPT.0000000000000099 27537070 PMC5158094

[27] Beck Jepsen D, Robinson K, Ogliari G, Montero-Odasso M, Kamkar N, Ryg J, et al. Predicting falls in older adults: An umbrella review of instruments assessing gait, balance, and functional mobility. BMC Geriatr. 2022;22(1):615. doi: 10.1186/s12877-022-03271-5 35879666 PMC9310405

[28] Pieruccini-Faria F, Sarquis-Adamson Y, Anton-Rodrigo I, Noguerón-García A, Bray NW, Camicioli R, et al. Mapping associations between gait decline and fall risk in mild cognitive impairment. J Am Geriatr Soc. 2020;68(3):576–84. doi: 10.1111/jgs.16265 31846071

[29] Segev-Jacubovski O, Herman T, Yogev-Seligmann G, Mirelman A, Giladi N, Hausdorff JM. The interplay between gait, falls and cognition: Can cognitive therapy reduce fall risk?. Expert Rev Neurother. 2011;11(7):1057–75. doi: 10.1586/ern.11.69 21721921 PMC3163836

[30] Montero-Odasso M, Speechley M. Falls in cognitively impaired older adults: implications for Risk Assessment And Prevention. J Am Geriatr Soc. 2018;66(2):367–75. doi: 10.1111/jgs.15219 29318592

