# Supplementary material for: Validation of a portable pressure tile system for standing and stepping stability assessment in young and older adults
Source: PLoS One. 2026 Jul 1;21(7):e0351180. doi: 10.1371/journal.pone.0351180 (PMC13322508; doi:10.1371/journal.pone.0351180)
Supplement: Attachment — Submitted filename: Response letter PONE-D-26-00279_R3.docx [file pone.0351180.s002.docx]

**Response to Review**

We thank the Editor and Reviewers for their careful consideration of our work, and we appreciate the helpful suggestions for improving our manuscript. Please find below a point-by-point response to the Editor’s requested revisions followed by responses to the Reviewers’ critiques. We include the page and line numbers where the changes can be found in the TrackChanges version of the revised manuscript.

Please note the following changes made in the process of revising the manuscript that were not specifically requested:

1. In the title we have removed the hyphen between the words “pressure” and “tile”.
2. In Table 3 we replaced the grouping label for the StepScan tile from SS to PT and only refer to it as pressure tile versus force plate. The reason for the change is that the label SS had previously been defined for Static Standing.
3. We replaced the text “StepScan™” with “pressure tile” in various places (marked as changes) where it made sense to use generalized terms for the pressure tiles and force plates.

Please also note that data for the project have been deposited for the present study and the CMBEC-47 conference paper. The following is a temporary link to view the datasets.

<https://dataverse.lib.unb.ca/previewurl.xhtml?token=974da41b-454e-419a-bb71-81a40aa65403>

**Editor**

1. Please ensure that your manuscript meets PLOS ONE's style requirements, including those for file naming. The PLOS ONE style templates can be found at

https://journals.plos.org/plosone/s/file?id=wjVg/PLOSOne_formatting_sample_main_body.pdf and

https://journals.plos.org/plosone/s/file?id=ba62/PLOSOne_formatting_sample_title_authors_affiliations.pdf.

*Response*: We have double checked our manuscript and believe it follows the example format.

2. We noted in your submission details that a portion of your manuscript may have been presented or published elsewhere. [A portion of the study was presented at the Canadian Medical and Biological Engineering Society conference in Fredericton, NB, May 2025.] Please clarify whether this [conference proceeding or publication] was peer-reviewed and formally published. If this work was previously peer-reviewed and published, in the cover letter please provide the reason that this work does not constitute dual publication and should be included in the current manuscript.

*Response*: The CMBEC-47 conference paper was peer reviewed and published in the conference proceedings. A copy of the 2-pg conference paper has been uploaded to the manuscript files and a description of that preliminary study has been added to the Introduction. That study examined raw CoP X-Y coordinate time series (trajectories) for the young cohort of 15 and did not examine stability parameters or age effects. Data overlap would only be the raw outputs from the tiles and force plates for the young cohort whose data was used in both studies. Also note that Figure captions now cite the CMBEC Conf paper (“Adapted from (10).”), as per CC0 1.0.

(<https://proceedings.cmbes.ca/index.php/proceedings/article/view/1247/1094>)

The copyright notice for CMBEC proceedings is pasted below

3. Please be informed that funding information should not appear in the Acknowledgments section or other areas of your manuscript. We will only publish funding information present in the Funding Statement section of the online submission form. Please remove any funding-related text from the manuscript.

*Response*: Funding statement has been removed from the main body of the manuscript.

4. If the reviewer comments include a recommendation to cite specific previously published works, please review and evaluate these publications to determine whether they are relevant and should be cited. There is no requirement to cite these works unless the editor has indicated otherwise.

*Response*: N/A

5. Please review your reference list to ensure that it is complete and correct. If you have cited papers that have been retracted, please include the rationale for doing so in the manuscript text, or remove these references and replace them with relevant current references. Any changes to the reference list should be mentioned in the rebuttal letter that accompanies your revised manuscript. If you need to cite a retracted article, indicate the article’s retracted status in the References list and also include a citation and full reference for the retraction notice.

*Response*: Our CMBEC Conference paper has been added as a reference (10).

**Reviewer #1**

This manuscript presents a validation study of a portable pressure-tile system for assessing balance and stepping stability, using AMTI in-floor force plates as a reference standard and evaluating sensitivity to age-related differences. The authors do a nice job of stating the clinical relevance of this study.

*Response*: We thank the reviewer for their positive response.

The study design is generally sound, however there are some suggestions to offer to help create a more suitable manuscript for publication.

One of my main concerns is that the authors continue to describe this measurement procedure as being meaningful for detecting falls risk, however the subjects that were included were healthy and likely not generalizable to a falls risk population. The authors should have at least performed a formal screening for falls risk, such that the they [sic] can classify the participants falls risk.

*Response*: This is a fair point to raise, as the Intro does focus heavily on falls. Future deployment of the StepScan technology for community falls prevention in older adults was indeed one of the motivating factors for this project, but it was not the purpose of this present study. To date there have been no independent validation studies of the StepScan system as a measurement tool for assessing mobility and balance, for any population. Validating the StepScan tiles with force plates will allow clinicians and researchers to use the technology in the field (homes, clinics, nursing homes, etc.) and know to what extent they can trust the outcomes.

To address the reviewer’s concern, we moved the first paragraph of the Introduction to the first paragraph of the Discussion (see **Pg 17-18, line 323-334**), and likewise, adapted the first paragraph of the original Discussion (more general in application) to be the opening paragraph of the Introduction (see **Pg 3, line 42-52**). This should help reader to focus attention on the study’s primary goal of measurement validation rather than its ability to predict future events, which will hopefully be follow-up studies from ours.

Line 101 indicates that the authors used the “Get Active Questionnaire” to screen potential volunteers, however they do not elaborate on the contents of this assessment, nor do they discuss what scores would be required in order to qualify for study participation. This should be clarified in resubmission.

*Response*: The GAQ is a common screening questionnaire used in studies where physical exertion is required. It is not a quantitative tool (there is no “score” per se). Rather it has filter questions aimed at determining if the participant has any medical or other conditions that would contraindicate moderate intensity exercise. The Step-Up/Step-Down test would classify as moderate intensity for most adults and thus was considered a safety measure to ensure all volunteers could participate at a minimal risk level. We have clarified this point in the Methods (see **Pg 6, Lines 108-110**).

I understand that the threshold of measurement accuracy of 5mm was utilized based upon the StepScan, however it is not clear that this can be reasonably extrapolated to the ability to identify clinically relevant differences between those at risk of falls. The authors state the measurement differences ranged from 1-3mm on average. The clinical utility of these differences should be commented on in the discussion.

*Response*: To be clear, the 1-3mm differences being referred to are the differences between measurement methods (StepScan versus AMTI) and not the effect sizes relevant to detecting to mobility and balance decline. Current StepScan tiles will always have a limitation of 5mm precision. Our study shows that the accuracy is essentially a “pixel” (within 5mm) when one is considering relative metrics such as CoP-based parameterization. This information will also allow researchers to design experiments with the threshold of accuracy in mind when powering studies based off minimally important clinical differences (which may be >> 5mm). We expand upon this in the Discussion (see **Pg 18-19, Lines 345-350**).

Line 202 – What type of ICC was utilized?

*Response*: In SPSS, the ICC was for Absolute Agreement using a two-way mixed ANOVA with averaged measures, which is equivalent to the Shrout & Fleiss ICC(2,k) model. We now include this in the Methods (see **Pg 11, Line 214**) as well as the Abstract.

Reporting:

The authors should include representative Bland–Altman plots and provide a clearer interpretation of limits of agreement in relation to clinically meaningful thresholds.

*Response*: We now include Bland-Altman plots for key measures. See new **Fig. 3**.

**Reviewer #2**

The paper presents a mixed validation approach to evaluate a commercially available portable pressure tile system in terms of measurement accuracy. To this end, a StepScan™ pressure tile system was placed upon in-floor mounted AMTI™ force platforms for simultaneous data acquisition.

The aim is to assess if the stability parameters derived from pressure tiles agree with those derived from the industry “gold standard” (in-floor) force plates. In addition, they analyzed whether these parameters are sufficiently sensitive to detect age-related decline in balance.

The study is well described and the results presented appropriately.

*Response*: We thank the reviewer for their positive response.

The experimental setup configurations are well described in the text. However, the use of the wooden box to support the StepScan™ pressure tile should be better explained in relation to its interference in obtaining the center of pressure (CoP) on the force platform on which it is supported.

Wouldn't a solid wooden box supported on the force platform distribute the pressure of the footstep over the entire area of the platform, altering the results obtained from the CoP variation?

Could the results be compared in the same way with the force platform on which the StepScan™ is directly supported?

*Response*: The force plate CoP is based on the forces sensed in the 3-axis load cells at each of the four corners of the plate and their moments relative to the point of load application on the plate surface. The plywood box (also with a flat bottom) sitting upon the plates would (if non-moving and rigid) have the same effect as putting the tile directly on the plate. In data processing, the only requirement is to set the height of the contact surface relative to the force plate surface, which projects the CoP measurement from the FP to the tile surface along the line of vertical ground reaction force. Although not discussed in detail in the manuscript, we validated graphically that the CoP was being properly transformed into contact surface coordinates for both the low tile and the high tile on the box. We have added some additional text to the Methods to clarify (see **Pg 6, Line 118-122**).

Table 2 presents the characteristics of the participants in the two groups described, including body weight. Based on the standard deviation shown, a reasonable difference in weight can be observed between participants. The authors could include a column with the participants' Body Mass Index, as well as a brief discussion of how this parameter can affect the analysis of the participants' body balance.

*Response*: We have added BMI to Table 2. The between-groups comparison was similar as for height and weight alone – there was no statistically significant difference between the young (23.1+/-3.1 kg/m^2) and old (25.4+/-4.4 kg/m^2) groups (p=.114). Nevertheless, larger samples may show differences, and so we do address these issues in our new Limitations section (see **Pg 20, Lines 375-378**).

The authors first used the abbreviation CoM in the text on line 181, but did not indicate what it stood for (Center of Mass).

Review repeated words in the text, e.g. lines 69 and 252.

*Response*: We thank the reviewer for catching these typos.
